# Enhancing Lithium Monitoring: A Comparative Analysis of Saliva, Erythrocyte, and Plasma Levels in Psychiatric Patients

**DOI:** 10.1192/j.eurpsy.2025.1094

**Published:** 2025-08-26

**Authors:** D. Vlahović, D. Karlović, V. Peitl, Z. Karlović

**Affiliations:** 1 Department of Psychiatry, Sestre milosrdnice University Hospital Center; 2 Catholic University of Croatia; 3 Department of Endodontics and Restorative Dentistry, School of Dental Medicine, Zagreb, Croatia

## Abstract

**Introduction:**

Lithium is a key treatment for bipolar affective disorder, effectively managing mania, depression, and reducing suicidality. Due to its narrow therapeutic range, regular monitoring of plasma levels is essential to avoid toxicity. However, blood plasma testing can be costly and inconvenient. Non-invasive alternatives, such as saliva testing, have been explored, but with inconsistent results. Previous studies have shown discrepancies in saliva collection and storage, variations in processing methods, and differences in lithium detection techniques. Recent research suggests that erythrocyte lithium levels may better reflect brain concentrations and predict treatment response. This study investigates the correlation between lithium concentrations in different types of biological media, aiming to find a more convenient and effective monitoring method for patients.

**Objectives:**

This study aimed to examine the correlation between lithium concentrations in saliva, plasma, and erythrocytes at multiple daily time points in patients undergoing lithium carbonate treatment.

**Methods:**

A total of 77 patients were recruited from the Clinical Department of Psychiatry, Sestre Milosrdnice University Hospital Center, Zagreb, Croatia, between January and August 2024. Participants included inpatients and day hospital patients diagnosed with bipolar affective disorder, treatment-resistant depressive disorder, or depressive disorder with a high suicide risk. All participants were newly initiated on lithium carbonate therapy, receiving 300 mg at 9:00 and 21:00 for 5 days. Exclusion criteria included conditions affecting salivation, pregnancy, and medications that alter lithium levels. Lithium concentrations in saliva, plasma, and erythrocytes were collected at 9:00, 10:00, 11:00, 12:00, 15:00, and 20:00. Samples were processed and analyzed using a spectrophotometric method.

**Results:**

To assess the correlation between lithium concentrations in saliva, plasma, and erythrocytes, linear regression analysis was conducted. The results indicated a statistically significant correlation between lithium levels in saliva and plasma (p<0.01). However, no significant correlations were observed between lithium concentrations in saliva and erythrocytes (p>0.01) or between plasma and erythrocytes (p>0.01).

**Conclusions:**

Aforementioned finding suggest that saliva could be used for lithium monitoring. Utilizing saliva could significantly enhance treatment options for patients with mood disorders while contributing to treatment efficacy, patient safety, and adherence.

**Disclosure of Interest:**

None Declared

